# Between Awareness and Readiness: Perceptions of Artificial Intelligence Among Indonesian Mental Health Professionals

**DOI:** 10.3390/bs16091622

**Published:** 2026-09-10

**Authors:** Fransiska Kaligis, Alifa Rahma Rizqina, Billy Pramatirta, Natasha Vania Theresia Purba, Farah Nabila Widyaputri

**Affiliations:** 1 Department of Psychiatry, Faculty of Medicine, Universitas Indonesia, Depok 16424, Indonesia; 2 Cipto Mangunkusumo Hospital, Jakarta 10430, Indonesia; 3 Medical Staff Group of Psychiatry, Universitas Indonesia Hospital, Depok 16424, Indonesia; 4 Faculty of Medicine, Universitas Indonesia, Depok 16424, Indonesia

**Keywords:** artificial intelligence, health personnel, psychiatric services, mental health professionals

## Abstract

Background: Artificial Intelligence (AI) is increasingly used in mental health practice, yet evidence on healthcare professionals’ knowledge and perception regarding its use remains limited. This study evaluated Indonesian mental health professionals’ perceptions of AI use in psychiatric services. Methods: A cross-sectional study was conducted among 267 healthcare professionals across Indonesia, using the Shinners Artificial Intelligence Perception (SHAIP) questionnaire. Results: All participants (100%) reported awareness of AI, and 53.2% had already applied it in daily practice. Participants aged 41–50 y.o. (compared to ≤40 y.o., *p* < 0.01) and profession (general practitioner and resident, compared to psychiatrist, each *p* < 0.01), were significantly associated with AI use after multivariable adjustment. Overall perception of AI was positive. Perceived professional impact differed significantly by profession (resident vs. psychiatrist, *p* = 0.03), and by workplace setting (psychiatry hospital vs. general hospital, *p* = 0.02) after multivariate analysis. Despite this generally positive perception, 82.8% of participants reported that they received no training in AI use, while only 21.0% agreed that they had received adequate formal AI training specific to their roles. Conclusion: Most Indonesian mental health professionals are aware of and already using AI in daily practice, with generally positive perceptions of its role. However, the gap between AI adoption and formal training calls for structured AI education in this workforce.

## 1. Introduction

Artificial Intelligence (AI) application in healthcare has become a major research interest in the past decade. In practice, AI encompasses a broad context, such as machine learning, natural language processing (NLP), rule-based expert systems, and robotic process automation (Davenport & Kalakota, 2019). The integration of AI in healthcare aims to improve service quality by increasing efficiency in processing, prediction, and resource allocation for individualized care (Dehbozorgi et al., 2025; Thakkar et al., 2024).

The World Health Organization (WHO) proposes a framework in which AI should be used as a partner, with the final responsibility and decision still governed by healthcare professionals; therefore, it is not a substitute for clinical expertise in daily practice (World Health Organization, 2021). Nevertheless, there are still some concerns surrounding the integration of AI into healthcare, including ethical and moral dilemmas, the impact on service quality, job security, and legal implications (Rony et al., 2024). One of the barriers for healthcare professionals is limited digital literacy for using AI in daily clinical practice, limiting its usage in clinical practice (Patel et al., 2026; Tegegne et al., 2023).

AI-based tools offer a distinct contribution to mental health services through their capacity to process large volumes of complex clinical and behavioral data to identify patterns that may not be apparent through conventional assessment (Olawade et al., 2024). Previous AI-based tools have demonstrated adequate accuracy in detecting, classifying, and predicting mental health disorders; assessing therapy response; and monitoring prognosis (Cruz-Gonzalez et al., 2025). In addition, AI-based mental intervention, through website-based therapy and digital applications, also increases access for patients and provides individualized services (D’Alfonso, 2020). Furthermore, AI-based robots and smartphone applications have also been trialed as therapeutic interventions for autism spectrum disorder and ADHD (Till & Briganti, 2023).

Research surrounding the use of AI in mental health services has been evolving rapidly, yet the adoption itself has outrun validated training, which high risk to patient safety. The use of AI in mental health services carries a meaningful risk of misuse, misdiagnosis, and privacy compromise. Systematic reviews have raised concern that large language model (LLM) tools such as ChatGPT may misdiagnose people with mental health disorders (Rahman et al., 2025; Yang et al., 2025). This further underlines the urgency for developers to prompt safeguards and instructions into these systems specifically for handling health-related conversations.

Understanding healthcare professionals’ knowledge and perception, as key stakeholders, is therefore essential. Prior research shows this perception varies widely depending on factors such as technological literacy, level of education, and personal experience with such tools (de la Fuente Tambo et al., 2025; Patel et al., 2026). Moreover, evidence on how mental health professionals perceive their use, including trust, perceived preparedness, and views on clinical accountability, remains limited. To date, we identified no published study examining these perceptions among Indonesian mental health professionals. This study addresses that gap by evaluating perceptions of AI use in psychiatric services among mental health professionals across Indonesia.

## 2. Materials and Methods

### 2.1. Study Design

The study is an analytical observational study with a cross-sectional design. Primary data were collected through an online questionnaire administered between February and March 2026.

### 2.2. Sample Recruitment

Participants were recruited using non-probability sampling, targeting Indonesian mental health professionals (psychiatrists, psychologists, general practitioners, etc.) who were actively engaged in clinical practice. A link to the online survey platform, containing study information, an informed consent form, a demographic questionnaire, and the main survey instrument, was disseminated to private and governmental hospitals and healthcare centers across Indonesia via email. Individuals who did not consent to participate or who submitted incomplete responses were excluded from the final analysis.

To preserve participant confidentiality, the dataset used in this study is not shared publicly, and no identifiable participant information is included in this article. A de-identified version of the dataset is available upon reasonable request to the corresponding author.

### 2.3. Data Collection

Questionnaires were distributed to participants via an online survey link. Participants who satisfied the eligibility criteria and agreed to participate were asked to complete a form comprising three sections. The first section captured identification and sociodemographic information, including initials, age, gender, profession, workplace, duration of workplace experience, and place of residence. The second section consisted of four items assessing knowledge and utilization of AI: (i) Do you know what Artificial Intelligence is? (ii) Are you currently using Artificial Intelligence (AI) in your clinical practice? (iii) Do you know about Artificial Intelligence (AI) use in mental health practice? (iv) Have you received any courses or training about Artificial Intelligence use in daily practice?

The final section of the form used the Indonesian language adaptation of the Shinners Artificial Intelligence Perception (SHAIP) (Shinners et al., 2022). The SHAIP is an instrument developed through a Delphi study designed to explore the perception of healthcare professionals on the use of AI. It consists of 10 items assessed using a 5-point Likert scale (1: strongly disagree, 5: strongly agree). Perception of AI use was categorized into two dimensions, which are the professional impact of AI (items 1–5 and 7) and preparedness for AI (items 6, 8–10). To ensure linguistic equivalence and cultural applicability within the Indonesian context, a rigorous translation and back-translation procedure was followed, adhering to the standard guidelines for cross-cultural adaptation of self-report measures (Beaton et al., 2000). First, two bilingual psychiatrists translated the original scale into Indonesian language independently. A synthesized version was then agreed upon after resolving minor discrepancies. Second, two independent translators (blind to the original scale) back-translated the synthesized version into English. Semantic equivalence was verified by comparing the back-translation with the original source. Third, an expert panel—comprising a psychiatrist, medical doctor, psychologist, and nurse—reviewed all translated versions alongside the original instrument. This committee systematically evaluated semantic, idiomatic, and conceptual equivalence to resolve any cross-cultural ambiguities, thereby generating the pre-final Indonesian version. Finally, cognitive interviews were conducted with a purposive sample of 10 health workers from the target demographic to pilot test the tool. This step aimed to ensure that the target population comprehended the items exactly as intended.

In the present study, the Indonesian adaptation of SHAIP has been validated. Content validity testing by six mental health professionals revealed that the I-CVI (Content Validity Index for Items) and CVR (Content Validity Ratio) values of the Indonesian version of the SHAIP questionnaire yielded significant results (>0.79). The average content validity ratio was 0.90, and the S-CVI/Ave value was 0.95, indicating good validity of the instruments. The Indonesian version of SHAIP also demonstrated acceptable reliability for the professional impact dimension (α = 0.710) with relatively lower internal consistency for the preparedness dimension (α = 0.610), comparable to the original SHAIP questionnaire (α = 0.832 for professional impact of AI and α = 0.632 for preparedness for AI).

### 2.4. Measured Outcomes

The primary outcome measure of this study was the perception of AI, represented by the two dimensions from the SHAIP questionnaire: the professional impact of AI and preparedness for AI.

### 2.5. Study Ethics

The study received full ethical approval from the Health Research Ethics Committee, Faculty of Medicine, Universitas Indonesia, on 12 January 2026, with the protocol number KET-48/UN2.F1/ETIK/PPM.00.02/2026.

### 2.6. Data Analysis

The Statistical Package for Social Science (SPSS) program, version 20, was used to analyze data in this study. Sociodemographic traits, knowledge, and AI use, as well as the distribution of SHAIP questionnaire responses, were examined descriptively. Categorical data will be presented as percentages (%), while numerical data will be presented in accordance with the distribution (using mean and standard deviation if the distribution was normal and median with interquartile range if the distribution was not normal).

The association between sociodemographic factors and knowledge and AI use was first analyzed using the chi-square test. Variables with *p* < 0.20 were included in the multivariate logistic regression model. To examine the associations between sociodemographic factors and perception of AI, independent samples *t*-tests were used for variables with two categories, and one-way ANOVA was applied for variables with more than two categories. Variables demonstrating a bivariate association with *p* < 0.20 were subsequently entered into a multivariate linear regression model to identify factors independently associated with perception of AI use.

## 3. Results

### 3.1. Participants’ Characteristics

A total of 267 participants were recruited in this study. Most of the participants were female (70.8%), with the median participant age of 36 years (range: 23–73), and 74.2% fell in the ≤40 years age group. Over half of the participants were married (62.9%). The distribution of professions in this study was psychiatrists (36.3%), followed by residents (26.6%), general practitioners (GP), and nurses (each representing 10.5%). Four final-year medical students (1.5%) who were at the psychiatry outpatient unit at the time of the survey also participated in the study. In Indonesia, medical students at this stage are exposed to patients and are directly engaged in mental health practice, under the supervision of the psychiatrist consultants. Although a final-year medical student is not officially recognized as a mental health professional, they often address and handle mental health issues in a clinical setting under supervision. We include their responses to provide additional insights from academics and individuals practicing within the mental health unit. Twenty-one (7.9%) other professionals (e.g., therapists, counselors) who consistently participated in mental health services were also included. Regarding workplace setting, 60.3% of participants were employed in general hospitals, and the majority reported ≤5 years of work experience (44.2%). Although most of the respondents were from Java (59.9%), there were representatives from every major island in Indonesia. This data is still proportional to the population of Java Island itself, which accounts for around 56% of the total Indonesian population (Badan Pusat Statistik, 2020). Full details of participants’ characteristics are presented in Table 1.

### 3.2. Knowledge and AI Use

Every participant knows the term Artificial Intelligence, with 53.2% currently using AI in their clinical practice (Table 2). In particular, more than half of participants (65.5%) know the application of AI in mental health practice. Despite this knowledge, most participants had not received any courses or training about AI use in daily practice (82.8%).

Table 3 illustrates the results of univariate analysis between demographic factors and the use of AI. Multivariate analysis was performed with purposeful selection. The logistic regression model was statistically significant (χ^2^ = 66.71, *p* < 0.01) and showed good fit (Hosmer–Lemeshow *p* = 0.98). The logistic regression model, including age group, profession, and work experience, showed that profession was the sole significant factor associated with AI use in clinical practice (*p* < 0.01) (Table 4). Compared to psychiatrists, general practitioners (*p* < 0.01, OR [95%CI]: 6.62 [2.17–20.19]), and residents (*p* < 0.01, OR [95%CI]: 5.54 [2.43–12.62]) had significantly higher odds of using AI. Age group as a whole was not significantly associated with AI use in the overall model (*p* = 0.06). However, when examined by specific category, participants aged 41–50 years old had significantly lower odds of using AI compared to younger participants (*p* = 0.01, OR [95%CI]: 0.28 [0.11–0.71]).

### 3.3. Perception of AI Use

Table 5 summarizes the answer distribution of each point from the SHAIP questionnaire. A significant proportion of participants believed that the use of AI could improve delivery of patient care (65.2%), improve clinical decision-making (48.7%), and improve population health outcomes in mental health services in Indonesia (59.9%). Almost half did not agree that AI will change their role as a healthcare professional in the future (44.6%), while 33.0% agreed with that statement. Regarding financial cost reduction, 37.8% of participants believed that the introduction of AI could yield such benefits, while 36.0% remained neutral on this topic.

Over half of the participants (51.3%) believed that overall healthcare professionals are prepared for the introduction of AI technology. The majority (62.5%) did not believe that AI may take over part of their role as a healthcare professional. Only 21.0% agreed that they had received adequate formal training to use AI specific to their roles, while 44.2% disagreed and 34.8% were neutral. More than half of participants believed that there is an ethical framework in place for the use of AI technology in their workplace (60.7%), and full responsibility lies with the healthcare professional whenever AI technology makes an error (65.5%).

### 3.4. Factors Associated with Perception of AI Use

Univariate analysis examining associations between demographic factors and perception of AI use, encompassing both professional impact and preparedness dimensions, is presented in Table 6. Practice location was significantly associated with different levels of perceived professional impact of AI. Tukey post hoc analysis revealed significant differences between psychiatric hospitals and other workplaces (university/school), as well as between clinics and other workplaces (university/school). No variables demonstrated a significant association with perceived preparedness for AI.

Multivariate analysis was conducted using purposeful selection (Table 7). For the professional impact dimension, the regression model included age, gender, profession, and practice location as predictors. The model was statistically significant (F(14, 252) = 2.26, *p* < 0.01); however, it only explained 6.2% of the variance (adjusted R^2^ = 0.062; R^2^ = 0.112). Male participants demonstrated a significantly higher perception score on professional impact of AI compared to females, with a mean difference of 0.21 points (95% CI: 0.06 to 0.37, *p* < 0.01). In terms of occupation, residents showed significantly higher perception scores on this dimension compared to psychiatrists with a mean difference of 0.21 points (95% CI: 0.02 to 0.40, *p* = 0.03). Additionally, healthcare workers employed at psychiatric hospitals demonstrated significantly lower professional impact perception scores compared to those working at the general hospital with a mean difference of −0.22 points (95% CI: −0.40 to −0.04, *p* = 0.02). For the preparedness dimension, no variables reached statistical significance in univariate analysis (all *p* ≥ 0.20; Table 6), and multivariate analysis was therefore not performed. This uniformity across demographic and professional subgroups is addressed further in the Discussion Section.

## 4. Discussion

### 4.1. Principal Findings

The integration of AI into healthcare, including mental healthcare services, has expanded substantially in recent years, emphasizing the need for healthcare professionals to develop awareness of how this technology can be incorporated into daily practice. Our study found that while all respondents reported general awareness of AI, only about two-thirds were familiar with its specific applications in mental health practice. Over half of participants (53.2%) had already incorporated AI into their clinical work. AI use differs by profession and years of experience, with more senior and specialist clinicians less likely to report using AI compared to younger and less experienced colleagues. The reasons for this pattern warrant further investigation, although existing evidence suggests greater exposure to AI is associated with increased likelihood of use (Heinrichs et al., 2025).

With respect to perceived professional impact, most participants agreed that AI could enhance patient care delivery, clinical decision-making, and population health outcomes. However, they did not perceive AI as likely to alter or replace their professional role in the future. Gender emerged as a factor associated with differences in this perception, with male participants reporting higher scores on professional impact of AI compared to female participants. Findings across previous studies regarding this relationship between gender and AI perception have been inconsistent. A Saudi Arabian study reported a similar direction, higher scores among males, but the difference was not statistically significant (Sharif et al., 2025). A study from Bahrain found no gender association at all (Al-Medfa et al., 2023), whereas a Syrian study reported significant male advantage (Swed et al., 2022). The role of women in society and their access to education should also be considered in these existing studies from Muslim countries. This inconsistency suggests that gender effects may be context-dependent rather than universal, potentially shaped by differences in sample size, specialty, healthcare system, or regional exposure to AI technologies.

Beyond gender, healthcare workers at psychiatric hospitals reported significantly lower perceived professional impact than those at general hospitals. Direct comparative studies examining AI perception by workplace setting remain limited, and existing evidence is mixed (Hoffman et al., 2024), possibly reflecting differences in health system structure and resource distribution between these sites. Perception also varied, although not significantly, by specialty and training level. This result aligns with a study in Bahrain which found psychiatrists to be highly skeptical of AI’s capacity for empathy or mental status examination, consistent with the discipline that relies on therapeutic alliance, longitudinal relationships, and clinical judgment grounded in interpersonal connection, which are the capacities AI does not have (Al-Medfa et al., 2023). Furthermore, the higher complexity of psychiatric analysis could contribute to a lower perceived professional impact of AI use in psychiatric hospitals.

Differences in AI perception have also been observed by specialty and training level, although the results were not significant. A qualitative interview study exploring general practitioners’ attitudes toward AI in Germany found that GPs generally held positive views, regarding AI as a promising tool for managing high patient volumes and reducing diagnostic errors. In contrast, a separate study surveying 791 psychiatrists from a developed country regarding perceived AI benefits and its potential to replace psychiatrists found that only 3.8% believed the technology would render their jobs obsolete, and 17% believed AI could replace human clinicians in providing empathetic care, further reinforcing the view that psychiatric professionals across settings tend to see AI as a supportive rather than substitutive tool (Doraiswamy et al., 2020).

Regarding preparedness for AI, more than half of the participants believed that overall healthcare professionals were prepared for the introduction of AI into their practice. Nonetheless, 82.8% reported having received no formal training or coursework on AI use in daily practice, and only one-fifth agreed that they had received training to use AI specific to their roles. Consistent with this, perceived preparedness did not differ significantly by any demographic examined, including age, sex, profession, workplace, or years of experience. Given that formal AI training was low across the entire sample, low preparedness appears to be a shared condition rather than one concentrated within any particular subgroup. This suggests that gaps in AI readiness among psychiatric healthcare professionals are more likely driven by systemic factors, such as the general absence of institutional training infrastructure, than by individual-level characteristics like age or clinical experience. This pattern has also been reported in Saudi Arabia and other countries (Sharif et al., 2025; Shinners et al., 2022), in which high general awareness was paired with limited training to support actual use. Without adequate training or support, AI may have real potential to unsafe practice and ethical dilemmas in daily clinical routine.

Some of the most common concerns for AI use in healthcare are regarding data privacy, algorithm transparency, and stakeholder involvement in the application of AI in mental health (Dehbozorgi et al., 2025). Specifically, mental health practices require heightened consideration, as the complexity of mental health disorders often involves holistic and comprehensive symptoms, environmental factors, and personal histories, which AI algorithms may struggle to accurately diagnose with nuanced interpretation. Moreover, the performance of the algorithm itself heavily depends on the quality and diversity of data they trained on, meaning that bias even in AI cannot be disregarded (Thakkar et al., 2024; Walsh et al., 2020). This highlights that human oversight is always crucial in terms of AI use in mental healthcare, a framework that is heavily proposed by the WHO and agreed by the participant.

### 4.2. Future Implications

The findings highlight an urgent need for targeted training to prepare clinicians for AI’s impact on the patient-provider relationship, a concern that carries particular weight in mental health care given how important the patient–physician relationship is to treatment itself. Bridging this literacy gap matters not only for operational efficiency, but for upholding ethical standards and patient safety in AI use. It also implies the importance of incorporating AI training into medical education in order to properly equip future mental healthcare professionals before these tools are integrated in practice.

### 4.3. Limitation of Study

This study has several limitations which should be taken into account. First, its cross-sectional design captures perceptions at a single point in time and cannot establish causality or explain how attitudes toward AI might shift. A qualitative approach through in-depth interviews or focus groups would be better suited to understand further regarding healthcare professionals’ perceptions and the reasoning behind these questionnaire answers. This study did not examine specific workplace conditions, such as institutional AI infrastructure, staffing levels, or resource allocation, that may explain the observed difference in perceived professional impact and preparedness for AI.

Subsequently, sample recruitment via email distribution would introduce selection bias and limit generalizability, as participants more familiar with technology (particularly younger respondents) may have been more likely to participate in the study. We also did not specify which AI tools the respondents had used for their clinical practice, which added to the methodological limitation of our study. Specifically, the questionnaire did not distinguish among different types of AI applications, such as generative AI, documentation or administrative tools, educational applications, or diagnostic and clinical decision-support systems. Clinicians’ perceptions may differ between administrative AI, which primarily supports workflow, and diagnostic or risk-prediction algorithms, which may directly influence clinical decision-making.

Additionally, the preparedness subscale of the Indonesian SHAIP questionnaire in this study demonstrated relatively low internal consistency (α = 0.610), below the conventional threshold of 0.70, consistent with the comparatively lower reliability of this subscale in the original SHAIP questionnaire as well (α = 0.632). This suggests that items within this subscale may not fully capture a unified construct, possibly reflecting a smaller number of items in this dimension. This may partly explain why no demographic or professional variables were significantly associated with preparedness scores, as the observed null association could reflect measurement imprecision rather than a genuine absence of relationship. Future research should incorporate these variables to determine whether this difference reflects setting-level resources or other factors in psychiatric practice itself.

Lastly, although the regression model was statistically significant, the low predictive power (11.2%) suggests that other unmeasured factors may largely contribute to perceptions of AI’s professional impact and should be explored in future research. Therefore, the findings should be interpreted cautiously and should not be considered a comprehensive explanation of professional impact perceptions of AI.

## 5. Conclusions

Most Indonesian mental health professionals are aware of and already using AI in daily practice with generally positive perceptions of its role. However, a substantial gap exists between adoption and formal training, which may affect patient safety and ethical practice. Structured AI training for this workforce is therefore a priority for mental healthcare professional development.

## Figures and Tables

**Table 1:** Demographic factors of study participants.

Demographic Factor	N (%)
Sex	
Male	78 (29.2%)
Female	189 (70.8%)
Age	Median (min–max): 36 (23–73)
≤40 years old	198 (74.2%)
41–50 years old	49 (18.4%)
51–60 years old	18 (6.7%)
>60 years old	2 (0.7%)
Marriage status	
Married	168 (62.9%)
Not married	90 (33.7%)
Divorced/widowed	9 (3.4%)
Profession	
Psychiatrist	97 (36.3%)
General practitioner	28 (10.5%)
Psychiatric resident	71 (26.6%)
Final-year medical student working in a mental health unit	4 (1.5%)
Nurse	28 (10.5%)
Psychologist	18 (6.7%)
Others (e.g., therapist, counselor)	21 (7.9%)
Workplace	
General hospital	161 (60.3%)
Psychiatry hospital	47 (17.6%)
Clinic	41 (15.3%)
Community health center (Puskesmas)	11 (4.1%)
Others (university, school)	7 (2.6%)
Work experience	
≤5 years	118 (44.2%)
6–10 years	58 (21.7%)
>10 years	91 (34.1%)
Place of residence	
Greater Jakarta	107 (40.1%)
Java (other than Greater Jakarta)	53 (19.8%)
Sumatera	37 (13.9%)
Kalimantan	6 (2.2%)
Sulawesi	48 (18.0%)
Bali, NTB, NTT	6 (2.2%)
Maluku	1 (0.4%)
Papua	9 (3.4%)

**Table 2:** Knowledge and AI use in mental health services in Indonesia.

Question	No	Yes
	N (%)	
Do you know what Artificial Intelligence is?	0 (0%)	267 (100%)
Are you currently using Artificial Intelligence (AI) in your clinical practice?	125 (46.8%)	142 (53.2%)
Do you know about Artificial Intelligence (AI) use in mental health practice?	92 (34.5%)	175 (65.5%)
Have you received any courses or training about Artificial Intelligence use in daily practice?	221 (82.8%)	46 (17.2%)

**Table 3:** AI use in mental health services in Indonesia.

Variable	Are You Currently Using AI in Your Clinical Practice?		*p*
	Yes	No	
Age group			<0.01 *
≤40 y.o. *	125 (63.1%)	73 (36.9%)	
41–50 y.o. **	10 (20.4%)	39 (79.6%)	
51–60 y.o.	6 (33.3%)	12 (66.7%)	
>60 y.o.	1 (50.0%)	1 (50.0%)	
Sex			0.50
Female	101 (53.4%)	88 (46.4%)	
Male	41 (52.6%)	37 (47.4%)	
Profession			<0.01 *
Psychiatrist *	34 (35.1%)	63 (64.9%)	
General practitioner **	23 (82.1%)	5 (17.9%)	
Resident **	56 (78.9%)	15 (21.1%)	
Nurse	11 (39.3%)	17 (60.7%)	
Medical student	3 (75.0%)	1 (25.0%)	
Psychologist	8 (44.4%)	10 (55.6%)	
Others	7 (33.3%)	14 (66.7%)	
Workplace			0.35
General hospital	93 (57.8%)	68 (42.2%)	
Psychiatry hospital	23 (48.9%)	24 (51.1%)	
Clinic	19 (46.3%)	22 (53.7%)	
Community health center (Puskesmas)	5 (45.5%)	6 (54.5%)	
Others (university, school)	2 (28.6%)	5 (71.4%)	
Work experience			0.01 *
≤5 years *	71 (60.2%)	47 (39.8%)	
6–10 years	34 (58.6%)	24 (41.4%)	
>10 years **	37 (40.7%)	54 (59.3%)	

* significantly more participants using AI in clinical practice. ** significantly fewer participants using AI in clinical practice. y.o. = years old.

**Table 4:** Logistic regression analysis of factors associated with AI use.

Demographic Factors	OR (95% CI)	Category *p*	Overall *p*
Age group (compared to ≤40 y.o.)			0.06
41–50 y.o.	0.28 (0.11–0.71)	0.01 *	
51–60 y.o.	0.56 (0.16–1.96)	0.37	
>60 y.o.	1.45 (0.08–25.96)	0.80	
Profession (compared to psychiatrist)			<0.01 *
General practitioner	6.62 (2.17–20.19)	<0.01 *	
Resident	5.54 (2.43–12.62)	<0.01 *	
Nurse	1.20 (0.48–3.01)	0.70	
Medical student	2.90 (0.28–29.71)	0.37	
Psychologist	2.44 (0.79–7.51)	0.12	
Others	0.53 (0.18–1.52)	0.24	
Work experience (compared to ≤5 years)			0.18
6–10 years	0.48 (0.21–1.06)	0.07	
>10 years	0.67 (0.30–1.46)	0.31	

Note. “Overall *p*” reflects the likelihood ratio test for each factor as a whole; “Category *p*” reflects the significance of the individual coefficient for that category relative to the reference group. y.o. = years old. * statistically significant.

**Table 5:** Distribution of perception of AI use according to the SHAIP questionnaire.

Questionnaire Item	Disagree Continuum	Neutral	Agree Continuum
	N (%)		
Item 1. I believe that the use of AI in my specialty could improve the delivery of patient care	14 (5.2%)	79 (29.6%)	174 (65.2%)
Item 2. I believe that the use of AI in my specialty could improve clinical decision-making	38 (14.2%)	99 (37.1%)	130 (48.7%)
Item 3. I believe that AI can improve population health outcomes	23 (8.6%)	84 (31.5%)	160 (59.9%)
Item 4. I believe that AI will change my role as a healthcare professional in the future	119 (44.6%)	60 (22.5%)	88 (33.0%)
Item 5. I believe that the introduction of AI will reduce the financial cost associated with my role	70 (26.2%)	96 (36.0%)	101 (37.8%)
Item 6. I believe that overall healthcare professionals are prepared for the introduction of AI technology	63 (23.6%)	67 (25.1%)	137 (51.3%)
Item 7. I believe that one day AI may take over part of my role as a healthcare professional	167 (62.5%)	55 (20.6%)	45 (16.9%)
Item 8. I believe that I have been adequately trained to use AI that is specific to my role.	118 (44.2%)	93 (34.8%)	56 (21.0%)
Item 9. I believe there is an ethical framework in place for the use of AI technology in my workplace	50 (18.7%)	55 (20.6%)	162 (60.7%)
Item 10. I believe that if AI technology makes an error, full responsibility lies with the healthcare professional	57 (21.4%)	35 (13.1%)	175 (65.5%)

Note: Answers for strongly disagree and disagree were combined into “disagree continuum”, while agree and strongly agree were combined into “agree continuum”.

**Table 6:** Demographic factors and perception of AI use.

Demographic Factors	Perception of Professional Impact on AI	Perception of Preparedness for AI
Age group	*p* = 0.140	*p* = 0.708
≤40 y.o.	3.22 (0.55)	3.31 (0.70)
41–50 y.o.	3.03 (0.59)	3.20 (0.59)
51–60 y.o.	3.07 (0.64)	3.38 (0.66)
>60 y.o.	3.17 (0.24)	3.25 (0.35)
Sex	*p* = 0.070	*p* = 0.739
Female	3.14 (0.48)	3.29 (0.68)
Male	3.28 (0.72)	3.32 (0.67)
Profession	*p* = 0.06	*p* = 0.26
Psychiatrist	3.09 (0.53)	3.16 (0.64)
General practitioner	3.30 (0.55)	3.45 (0.67)
Resident	3.27 (0.55)	3.40 (0.68)
Nurse	3.20 (0.68)	3.33 (0.77)
Medical student	3.42 (0.73)	3.56 (0.94)
Psychologist	2.92 (0.58)	3.33 (0.52)
Others	3.33 (0.54)	3.24 (0.74)
Workplace	*p* < 0.01	*p* = 0.38
General hospital	3.23 (0.55)	3.35 (0.70)
Psychiatry hospital	3.01 (0.56)	3.22 (0.62)
Clinic	3.06 (0.58)	3.18 (0.52)
Community health center (Puskesmas)	3.33 (0.32)	3.45 (0.51)
Others (university/school)	3.69 (0.77)	3.07 (1.22)
Work experience	*p* = 0.93	*p* = 0.67
≤5 years	3.18 (0.58)	3.26 (0.74)
6–10 years	3.20 (0.53)	3.30 (0.62)
>10 years	3.17 (0.56)	3.34 (0.62)

Note. Values are presented as Mean ± SD. *p*-values for each demographic factor represent an overall test of association between the factor and each perception dimension. y.o. = years old.

**Table 7:** Multiple regression analysis of factors associated with perception of professional impact on AI.

Demographic Factors	B (Unstandardized Coefficient) (95% CI)	*p*
Age group (compared to ≤40 y.o.)		
41–50 y.o.	−0.03 (−0.23–0.17)	0.78
51–60 y.o.	0.01 (−0.28–0.30)	0.93
>60 y.o.	0.10 (−0.67–0.88)	0.79
Gender (male)	0.21 (0.06–0.37)	0.01 *
Profession (compared to psychiatrist)		
General practitioner	0.24 (−0.02–0.49)	0.07
Resident	0.21 (0.02–0.40)	0.03 *
Nurse	0.10 (−0.14–0.33)	0.42
Medical student	0.24 (−0.31–0.80)	0.39
Psychologist	−0.22 (−0.56–0.12)	0.21
Others	0.16 (−0.19–0.50)	0.36
Workplace (compared to general hospital)		
Psychiatry hospital	−0.22 (−0.40 to −0.04)	0.02 *
Clinic	−0.08 (−0.32–0.15)	0.50
Community health center (Puskesmas)	0.09 (−0.32–0.50)	0.67
Others (university/school)	0.43 (−0.02–0.88)	0.06

Note. *p*-values represent individual regression coefficients from multivariate analysis, comparing each category to its reference group (indicated in parentheses), and are distinct from the overall univariate *p*-values reported in Table 6. y.o. = years old. * statistically significant.

## Data Availability

The data that support the findings of this study are available from the corresponding author upon reasonable request. The data are not publicly available due to privacy concern.

## Abbreviations

The following abbreviations are used in this manuscript:

AI	Artificial Intelligence
ADHD	Attention deficit hyperactivity disorder
GP	General practitioner
LLM	Large language model
NLP	Natural language processing
SHAIP	Shinners Artificial Intelligence Perception
WHO	World Health Organization
